# Consumer Cognition and Management Perspective on Express Packaging Pollution

**DOI:** 10.3390/ijerph19084895

**Published:** 2022-04-18

**Authors:** Sisi Wu, Xuan Gong, Yunfei Wang, Jian Cao

**Affiliations:** 1 School of Management, Zhejiang University of Technology, Hangzhou 310023, China; sswu_zjut@163.com (S.W.); gongxuan_up@163.com (X.G.); jcao@zjut.edu.cn (J.C.); 2 Center for Global & Regional Environmental Research, The University of Iowa, Iowa City, IA 52242, USA

**Keywords:** environmental pollution, consumer cognition, recyclable express packaging, green express packaging, government responsibility

## Abstract

Consumer awareness of environmental protection is getting stronger. However, with the development of the logistics industry, the environmental pollution caused by express packaging has become increasingly severe. Therefore, it is of great importance to know consumer cognition and willingness about how to reduce the express packaging pollution. In this study, through the analysis of 561 questionnaires, we analyze the impact of consumer evaluation of recyclable express packaging and green express packaging on responsibility awareness of government, logistics enterprises, and e-commerce corporates, and analyze whether there is a positive correlation between consumer evaluation and reducing environmental pressure. We find that consumers are willing to use recyclable express packaging and green express packaging, especially the latter. Moreover, the government is supposed to play a central role in solving environmental pollution problems caused by express packaging. It is recommended that the government proposes some corresponding solutions, such as introducing a packaging tax policy, setting up an environmental fund, and developing environment-friendly packaging materials. Meanwhile, consumers expect logistics enterprises and e-commerce companies to cooperate with the government actively and switch to using environment-friendly express packaging in a timely manner.

## 1. Introduction

In recent years, the public and government have been paying greater attention than ever to the boom of the logistics and express delivery industry in response to the rapid development of e-commerce platforms, such as Amazon, Taobao, eBay, etc. According to the statistics from State Post Bureau of the People’s Republic of China [1], the annual business income of express delivery industry in 2018 increased 21.8%, reaching 603.8 billion CNY (~$87.5 billion). The total number of express deliveries increased by 26.6% to 50.71 billion, which means the logistics enterprises needed to distribute 140 million express parcels daily. However, the data show that more than 60% of consumers will directly discard the express packaging after receiving the express [2,3]. The random disposal and overuse of express packaging are leading to increasingly serious environmental pollution [4]. In China, the severity of packaging waste pollution is second only to those of water pollution, marine pollution, and air pollution [5]. Therefore, it is of practical significance to study the consumer cognition on express packaging pollution, and explore solutions to related issues.

At present, the governments of many developed countries have taken measures to deal with the environmental pollution of express packaging materials, and promulgated a series of laws and regulations to regulate the recycling behavior of consumers. For example, Japan issued “Packaging Container Waste Recycling Law” and “Energy Conservation Law”, Germany also issued the “Packaging Ordinance” in 1991, and the United States stipulated the recycling of packaging waste in “Resource Conservation and Recovery Act”. Especially, some countries have specified both targets and requirements of packaging waste disposition [6]. On 11 February 2004, the European Union issued the Packaging and Packaging Waste Directive 2004/12/EC, which stipulated that the total recovery rate of packaging waste must reach 60% [7]. The instruction sets the specific recycling efficiency: about 60% of glass, over 60% of paper and paperboard, more than 50% metals, etc. [8]. In the same year, the recovery rate of cartons in Brazil reached 79%, and that of sterile packaging cartons and plastics was 22% and 21%, respectively [7]. 

Although there are detailed rules and regulations for the recycling of express packaging materials abroad, and more advanced garbage recycling methods have been established, the governments of developing countries pay less attention to this issue. In 2019, China officially began to implement the waste classification system, which can help consumers better classify express packaging, and effectively reduce environmental pollution. Regrettably, China has not issued laws and regulations specifically for pollution issues caused by packaging waste. Moreover, according to the data published by the State Post Bureau of China so far, less than 20% of express packaging in China is recycled, while the recovery rate of cardboard is below 50% and buffers are even 0% [9]. Thence, we hope to provide a reference for our government to formulate related policies for recycling express packaging and alleviating pollution problems. 

The commonly used traditional express packaging mainly consists of the following seven parts: paper-made cartons, waybills, envelopes, and plastic packaging bags, woven bags, tapes, and cushions. Polyethylene and polypropylene are the most commonly used raw materials for express packaging, which will cause environmental pollution [10]. For instance, harmful gases containing heavy metal particles are generated in the process of producing poor-quality plastic products in the factory [11]. When the concentration of such gases reaches a certain level, it will endanger human health. Further, inferior plastic packaging is often non-degradable, so that the soil and groundwater will be polluted due to improper landfills [8,12]. Additionally, if the express package is treated by incineration, a large amount of dioxin gas will be produced, which can cause infant deformity, cancer, and mutation [13]. Therefore, to achieve sustainable development in the express delivery industry and protect human health, we must switch to using environmentally friendly express packaging as soon as possible [14]. 

At present, the common environmental protection express packaging can be divided into two categories: recyclable express packaging and green express packaging. The former includes reusable cartons or plastic packaging, which will be recycled by logistics companies or e-commerce enterprises for many times until the packaging is damaged in protection function [8,15]. The latter refers to the packaging that meets the requirements of environment protection, which must remain harmless to the ecological balance and human health after being discarded (like landfilled or burned), and can be disposed directly as solid waste [16]. To be specific, green packaging in general has been used with fewer materials for saving resources to minimize the packaging waste, it can be naturally degradable [17], and is mostly made of environment-friendly raw materials: for example, degradable plastics, biodegradable plastics, compostable plastics, and bio-based plastics [18,19]. Therefore, the environmental pressure can be greatly relieved if the above two kinds of express packages are widely used.

As a member of developing countries, China is willing to continue to be the advocate and follower of global green economy, and keep improving the development ability of China’s green economy [20]. The use of environmental protection express packaging will become one of the new measures to protect the environment in China [16]. Zhejiang Province is one of the economically developed provinces and the most developed area of e-commerce industry in China, with a complete e-commerce supply chain, especially the express industry. Moreover, in Zhejiang, the people’s living standards are relatively high and the frequency and consumption amount of online shopping are relatively high, so correspondingly, more express packaging is used and received there. Therefore, taking Zhejiang as an example, we studied the attitude of Zhejiang consumers towards the environmental pollution caused by express packaging and roughly judged the views of Chinese consumers on this issue. 

In this paper, there are three main research goals. First of all, we hope to find out the key points about express packaging that consumers pay more attention to. Secondly, it is aimed to study whether consumer environmental awareness and evaluation of express packaging have an effective impact on the responsibilities division of governments, logistics enterprises, and e-commerce companies. Finally, we study the role of the government, logistics enterprises, and e-commerce companies in the prevention of environmental pollution by express packaging, and put forward opinions and suggestions on the rational use of express packaging for these three organizations, respectively. All in all, the purpose of this paper is to provide a reference for our government to solve the environmental pollution caused by express packaging, improve the contribution of enterprises in this problem, and find solutions for express packaging pollution, so as to help the logistics industry to achieve sustainable development.

The remainder of this paper is organized as follows. Section 2 reviews the related literature on express packaging choice and recycling. Section 3 presents the research methodology and data collection. Section 4 carries out basic analysis and results, including exploration factor analysis, descriptive statistical analysis, and cross-information analysis. The structural equation model and testing results are demonstrated in Section 5, and the discussion about the model is put in Section 6. Section 7 is the conclusions, recommendations, and future prospects.

## 2. Literature Review

A series of literature focuses on how to reduce express packaging waste. Express packaging is a type of household solid waste. Da Cruz et al. [6] and Roche Cerasi et al. [21] propose that express packaging can be reduced by classifying and recycling on a household basis. Some literature studied the factors that affect the recycling of express packaging, and proposed that public participation in recycling is the key to success [22,23,24]. Meng et al. [25] and Wang et al. [26] carried out research from the perspective of packaging recycling methods and recycling services, and their research shows that the convenient recycling method and perfect recovering services will effectively encourage consumers to participate in the classification and recycling activities of municipal solid waste. Sidique et al. [27] studied the impact of recycling prices on packaging recycling, and proposed that the establishment of a variable recovering price is an effective political demarche to promote recycling and reduce the generation of waste. De Weerdt et al. [28] discussed the effect of waste incineration taxation on industrial plastic waste generation through panel analysis. 

China is in the initial stage of express packaging recycling. A large number of scholars have studied the current situation of packaging recycling in China, and put forward corresponding improvement measures. Based on the perspective of low-carbon logistics, Wang et al. [29] analyzed the application status of express packaging recycling mode, and proposed that compared to the recovery rate, which reaches 50% to 60% in the developed countries, 20% in China is obviously weak. Zhang et al. [30] studied the recycling of express packaging based on sustainable development concept, and consider that the four reasons for the low volume of packaging recycling in China are the inadequate recycling policy, high recycling costs, poor awareness on environmental protection, and the lack of unified standards in the express industry. Xia et al. [2] and Yildiz-Geyhan et al. [31] investigated the measures to improve the recycling rate of express packaging, and mentioned that companies should avoid violent sorting, reduce the damage of express packaging, and take the initiative to recycle packaging. Fan et al. [11] focuses on the environmental impact of the rapid development of China’s express delivery industry, and proposed to reduce the environmental damage caused by express packaging by improving the recovery efficiency of express packaging, controlling the production sources of express packaging materials, promoting emerging technologies, and establishing a unified standard production model. 

Our research is also relevant to the literature studying consumers’ cognition of environmental protection and green purchasing intentions. Some countries have been carrying out long-term environmental protection education for the masses. Zhang et al. [32] mentioned that kindergartens can offer relevant courses to cultivate children’s awareness of environmental protection. Yamaguchi et al. [12] shows that economic instruments and related policies enforce consumers to purchase simple packaging products. Guo et al. [33] studied the impact of policies and regulations on consumers’ cognition of environmental protection, as well as the relationship between consumer environmental awareness and their behavior, and propose relevant measures to promote consumers to unify their consumption intention and behavior. Martinho [34] shows that the contribution of consumers to environmental protection is obviously related to their consumption preferences.

It has also been mentioned in some studies that those with stronger environmental beliefs are more likely to engage in environmentally-oriented purchases [35,36]. Hao et al. [16] analyzed the factors that affect consumers’ willingness to pay for green packaging, and show that most consumers have insufficient certain knowledge regarding green packaging, whereas they have a strong willingness to purchase. Ketelsen et al. [37] studied the consumers’ response to environmentally-friendly food packaging. Klaiman et al. [38] investigated consumer preferences and demand for packaging material and recyclability, and found that consumers’ willingness to buy green materials was significantly higher than that to buy recyclable materials in the United States. Different from the United States, Hao et al. [16] found that Chinese consumers do not have an obvious preference to packaging, and they are more susceptible to education, media campaigns, and environmental awareness in buying green products. 

A large amount of literature has carried out research on consumers’ recycling willingness and recycling behavior of packaging. Reijonen et al. [15] show that the consumers’ cognition of environmental protection, policies, and incentive mechanism have a significant impact on their recycling behavior. Antonopoulos et al. [8] point out that recycling slogans can be posted in public places to guide consumers to choose environmentally friendly packaging products. In terms of the consumer willingness of recycling, Lu et al. [39] and Xie et al. [40] have found that the broader the environmental protection propaganda and the more complete the social incentive mechanism, the higher their willingness to recycle. There is a close connection between recycling awareness and recycling behavior as well [34]. Kelly et al. [41] shows that the more widely consumers know about environmental protection, the more likely they are to actively adopt recyclable express packaging. Miliute-Plepiene et al. [42] mentioned that with the gradual maturity of the recycling system, consumers’ recycling awareness has a greater impact on recycling behavior.

After reviewing the above literature, we found that the existing research lacks the study on consumer cognition on express packaging choice, responsibility subjects of express packaging pollution and corresponding solutions. Therefore, we discussed the environmental pollution of express packaging from different angles. Through the study of consumers’ cognition on the characteristics of recyclable and green express packages, our research analyzes their understanding of the responsibilities of the government, logistics enterprises, and e-commerce enterprises, as well as the help of these understandings to alleviate environmental pressure. 

## 3. Research Methodology and Data Collection

Nowadays, China is facing the problem of environmental pollution caused by express packages, but consumers have not paid enough attention to this problem. This questionnaire is designed to help us understand consumers’ cognition of this problem by means of survey. The 20 questions of the questionnaire are divided into two parts: basic information and others (See Part S1 of Supplementary Material for the content of the questionnaire). At the beginning of the questionnaire, a screening question is set up to pick out the unqualified respondents. When the answer to that question is selected as “No” which means the respondents have not ever used express service, the questionnaire will be closed immediately.

The survey was first carried out in Zhejiang Province, which is located on the southeast coast of China and is a part of the Yangtze River Delta. The total area of Zhejiang Province is 40,733.8 square miles (as large as Iceland, slightly smaller than half of the UK) and the total population is 57.4 million (9.1 million less than the UK). It is composed of 11 cities. Hangzhou, Ningbo, and Wenzhou have relatively developed economic levels compared with other 8 cities.

The Likert five-level scale was used to evaluate the questions in our questionnaire. A pre-survey which includes 57 participants was used to avoid the ambiguity and increase the validity of the questionnaire. The initial questionnaire was revised and improved based on the opinions and suggestions provided by the participants, while the formal questionnaire covering all age groups in Zhejiang Province was released on 19 April 2019. 

**Figure 1:**
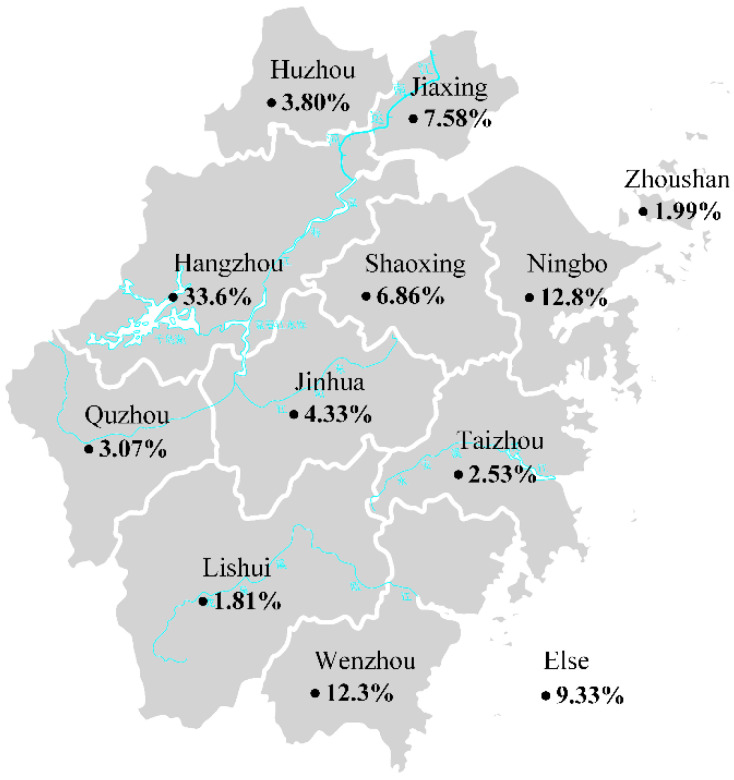
The region distribution of the questionnaire respondents.

Descriptive and exploratory research design effectively serves our research. A total of 600 survey samples were collected within two months after the questionnaire was sent out, which shows a positive response. Among them, 561 valid samples were obtained by eliminating all the invalid ones with the same answers, inconsistencies, or a large number of missing values. The recovery rate of the sample was 93.5% which is acceptable. The regional distribution of questionnaire sources is shown in Figure 1.

Our survey was conducted in Hangzhou and covered Zhejiang Province. The basic information of our research is presented in Table 1. As is shown in data results, the gender distribution of the respondents is relatively average, which is basically consistent with the gender ratio of China. The occupation of the respondents is manifold, including financial, real estate business, insurance, etc., the salary of them is mainly below $1094 (after tax per month), and the education background is between senior high school to undergraduate.

## 4. Basic Analysis and Results

### 4.1. Exploration Factor Analysis

An exploratory factor analysis of consumers’ views on the characteristics of recyclable express packaging (Q4: Do you care about the following characteristics of recyclable express packaging?) and green express packaging (Q7: Do you care about the following features of green express packaging?) was made by SPSS 22.0. The former includes reusable paper or plastic packaging, while the latter causes little or no harm to ecological environment and human health.

Referring to Shengliang et al. [43], the data from the questionnaire are suitable for exploratory factor analysis, supported by parameters of KMO coefficient, significance value and Cronbach’s α. As is shown in Table 2, KMO coefficient of Q4 is 0.901 (which is close to 1), with a significance is 0.000 (which is <<0.05) and Cronbach’s α is 0.876 (which is greater than 0.85), indicating that it is appropriate to use the factor analysis in Q4 because of the high level in reliability and validity of the data. For Q7, the three parameters mentioned above are 0.910, 0.000, and 0.880, respectively, which means the factor analysis can be used accordingly.

Three of the factors of “environmental protection and practicality”, “cost performance”, and “appearance” can be extracted from Q4, accounting for 75.6% of the total variance (see Part S2.1 of Supplementary Material for details). In general, the results of social science surveys are acceptable if the factors account for more than 70% [44]. In order to study the importance that consumers attach to these three factors, we conduct principal component analysis on Question 4, and obtain the principal component scores of the three factors (see Table 3), which are used as the basis for ranking importance. The letter y represents the principal component scores of each factor, that is, the importance of each factor. 

The result shows that “cost performance” is the most concerned factor of recyclable express packaging for consumers, followed by “appearance” and “environmental protection and practicality”. Therefore, we suggest that the performance–price ratio of express packaging materials should be taken as the reference factors for enterprises to design express packaging materials, such as price, reusability, and firmness. Based on the analysis of the result above, several feasible suggestions are put forward for packaging designers. In terms of cost performance, it is better to reduce the cost of recyclable packaging as much as possible. Meanwhile, the packaging ought to be more durable, so that it is not only reusable many times but can better protect internal goods. In the appearance aspect, distinguished from the ordinary packaging, the conspicuous one would catch consumers’ eyes and arouse their sense of recycling. Although consumers do not consider much about “environmental protection and practicability” for the time being, we still suggest that relevant factors should be pondered in the design of express packaging.

The three factors extracted from Q7 by the same method can still be categorized as “environmental protection and practicality”, “cost performance”, and “appearance” (see Part S2.2 of Supplementary Material for details). As shown in Table 4, the same method as for Question 4 is used to analyze the importance consumers attach to the three factors of green express packaging in the form of a principal component score. The letter y represents the value of the principal component score of different factors, that is, their respective importance.

According to the ranking in Table 4, the preference of consumers for green express packaging is the same as recyclable express packaging. The strongest preference for “cost performance” makes it necessary to fully consider the factors of price, reusability, and firmness when designing green express packaging. The factor of “appearance” ranks second. It is recommended that designers add some green elements when designing the appearance, such as using green as the main color or adding a green mascot. Beyond that, in order to match the green theme, the green express packaging can be designed to be different from the traditional or the recyclable ones [45]. Thus, consumers can quickly judge it as a green express package that will not pollute the environment by its appearance. Due to the less pressure on the environment caused by green express packaging (almost no environmental pollution), consumers are less concerned about the “environmental protection and practicability” factors.

### 4.2. Descriptive Statistics Analysis

The basic information of the respondents has been analyzed in Section 3, while the rest of the questionnaire will be studied in this chapter, including consumers’ understanding of the existing problems, awareness of the responsibility of the government, logistics enterprises and e-commerce enterprises, and the level of consumers’ willingness to protect the environment. 

The statistical results of the questionnaire show that 96% of the respondents have used express service. To enlarge these data to the total population of China, a large number of express packages are being put into use. With the boom of the express services industry and the surging of packaging, frequent use of express packaging may lead to a large amount of waste, which results in an increasing environmental pollution problem and brings huge pressure to the environment [13].

**Figure 2:**
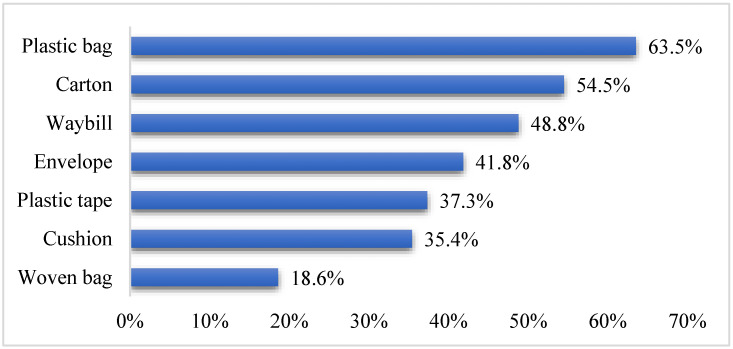
Ranking of seven types of express packaging discards.

The statistical results also show that, as is shown in Figure 2, consumers believe that the most (likely to be) wasted parts of the express packaging are plastic bags (63.5%), cartons (54.5%), and waybills (48.8%), while the woven bags (18.6%) with low utilization rate are less wasted. Envelopes (41.8%), tapes (37.3%), and cushions (35.4%) accounted for almost the same proportion. It means that consumers use plastic bags, cartons, and waybills more frequently in daily life. Thus, we can speculate that the usage of these three kinds of materials in express packaging is the largest.

In addition, the results of the survey show that 95.1% of the respondents are willing to use recyclable express packaging, while for the green express packaging, the respondent acceptance is slightly higher than the former, reaching 97.4%. The data show that consumer acceptance of recyclable express packaging and green express packaging are high, but consumers are relatively more willing to use disposable and environmentally friendly green express packaging. Therefore, it can be concluded that consumers are ready to accept environmental protection express packaging. Note that the environmental protection express packaging mentioned in this paper is a general term, covering recyclable express packaging and green express packaging. To sum up, we hope that logistics enterprises can improve their environmental protection level and gradually replace the non-degradable, non-landfill, and non-combustible traditional express packaging materials with environmental protection express packaging materials [18,19].

**Figure 3:**
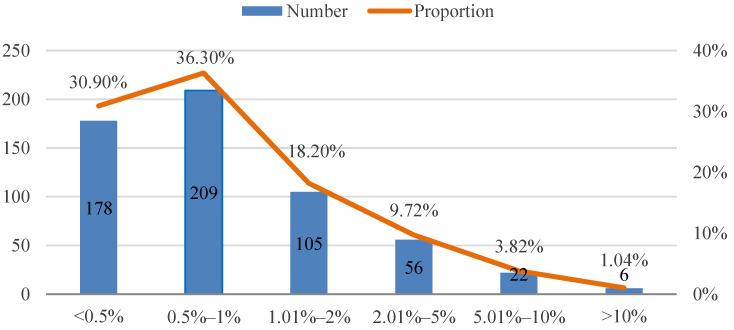
The premium range consumers are willing to pay for green express packaging.

Nnorom et al. [44] pointed out that customers are willing to pay a premium for green express packaging. Next, we studied the premium range consumers are willing to pay for green express packaging. As Figure 3 shows, it is acceptable for 85.4% of the respondents to pay less than 2% extra for using green express packaging. Among them, 18.2% of the respondents will accept 1–2% extra fees, 36.3% prefer the premium between 0.5% and 1%, while the remaining 30.9% hope the extra cost is less than 0.5%. Moreover, 1.04% of the respondents expressed their willingness to pay more than 10% of the additional fees. Therefore, we can speculate that some customers are indeed willing to pay a premium for green express packaging, and our survey provides a realistic reference for the pricing of green express packaging.

**Figure 4:**
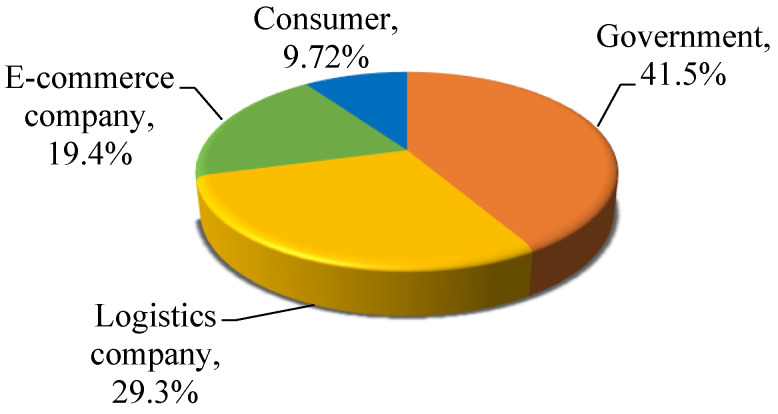
Consumer cognition on the responsible subject for reducing express packaging pollution.

Some literature studied how to reduce or even solve the environmental pollution caused by express packaging [12,33]. According to the analysis of the answer to Question 10 (Who should take the responsibility for managing or reducing the environmental pollution caused by express packaging?), we investigated the consumers’ awareness of the responsible subject for reducing express packaging pollution. The survey shows that about half of the respondents take the attitude that it is up to the government to call on the public to use recyclable and green express packaging. In addition, 29.3% and 19.4%, respectively, think that logistics companies and e-commerce enterprises should take the lead. Only 9.72% consider that it ought to be dominated by consumers, as shown in the following Figure 4.

According to our survey, we found that most consumers support the government’s measures to deal with the environmental pollution caused by express packaging. Further, we investigated what consumers think the government should do. As shown in Table 5, 74.9% of the consumers believe that corresponding tax policies should be promulgated in time to alleviate the environmental pollution of express packaging materials, while 6.25% of them hold a negative attitude and 18.9% of them are uncertain. The idea of setting up a fund for the environment received a 74.8% support. The fund is used to sponsor enterprises to establish a scientific recycling system, so as to alleviate environmental pollution of express packaging. The majority (75.3%) of the consumers agree with the view that the government ought to encourage scientific research institutions to promote the research of environment-friendly packaging materials, while 5.55% of them hold a negative attitude. In addition, more than 70% of the consumers also hope that the government would popularize the action of recycling express packaging.

In addition, Figure 4 also shows that more than 25% of the respondents hold the view that it is logistics companies’ responsibility to reduce the pollution of express packaging. Further, we surveyed what responsibility the consumers think the logistics enterprises should take. As is shown in Table 6, 76.9% of them suggest logistics enterprises to use paper tape instead of adhesive tape. More than 75% of respondents deem that door-to-door recycling services of express packaging provided by logistics companies will alleviate packaging pollution problems. Most respondents (73.6%) believe that logistics companies should guide customers to use recyclable or green express packaging, and 77.1% of them suggest logistics companies ought to establish an information system in recycling express packaging to improve recycling efficiency.

Another view is that e-commerce companies should bear the main responsibility for reducing the pollution of express packaging [40], which is supported by 19.4% of the respondents. Further, we investigated what consumers think the e-commerce corporate should take to reduce express packaging pollution. As shown in Table 7, 76.1% of the respondents consider that there will be a positive effect on alleviating environmental pollution if leading e-commerce enterprises choose recyclable or green express packaging. From this point of view, 6.08% are skeptical and 17.9% are uncertain. About 77.3% of respondents regard that e-commerce companies should establish recycling cooperation with logistics enterprises to jointly solve the packaging pollution problem. Only 4.52% of them believe that e-commerce companies have no obligation to motivate consumers to take part in express packaging recycling. In their opinion, recycling incentives cannot relieve environmental stress at all.

**Figure 5:**
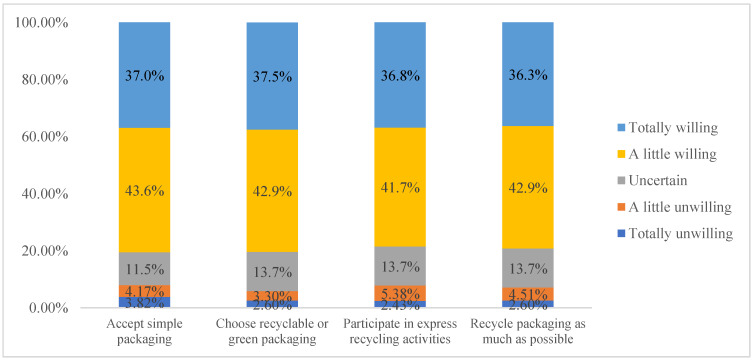
Consumers’ willingness to protect the environment.

There are two assumptions of Question 9 (If China’s recycling mechanism is increasingly perfect, and green express packaging is widely used, do you agree with the following behaviors?). One is that the recycling system and mechanism of express packaging have been complete, while the other is that recyclable and green express packaging have been put into use already. Under this premise, four aspects are designed to investigate the willingness of consumers to solve the environmental pollution problem caused by express packaging. As is shown in Figure 5, 80.6% of respondents prefer simple express packaging while 7.99% are unwilling to do so. The formers may hold the view that under the premise of full protection of inner products, the fewer packaging materials that are used, the less pollution there will be [5,17]. More than 80% of respondents choose recyclable or green express packaging actively. Further, they may express the view that traditional non-environment-friendly express packaging should be replaced by the recyclable and green one, so as to save resources and protect the environment [32,38]. More than three-quarters of the respondents express their willingness to recycle the express packaging as much as possible and put the environmental protection into practice.

### 4.3. Cross-Information Analysis

Cross-analysis is an extension of descriptive statistical analysis, which shows the relationships between variables. In this section, we focus on the relationship between two factors (city and gender) and other factors.

#### 4.3.1. Intersection between the Urban and Other Factors

**Figure 6:**
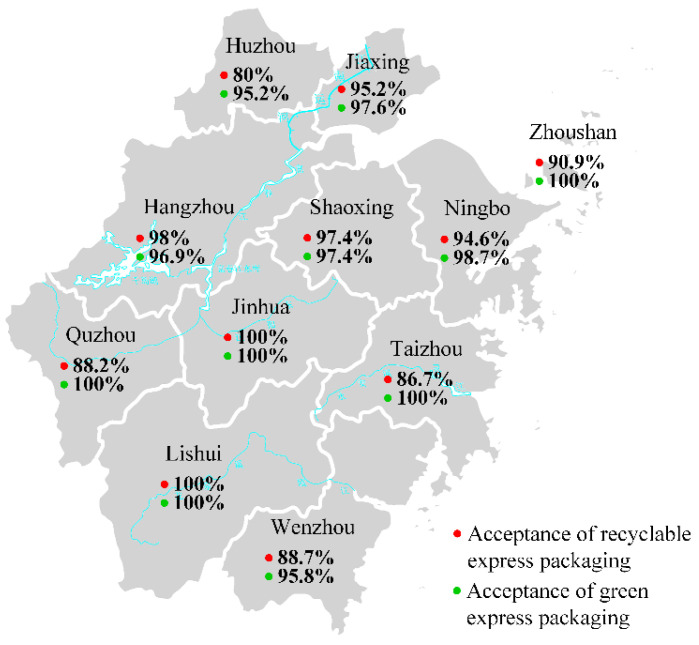
Consumers preferences for environment-friendly packaging in different cities.

As shown in Figure 6, survey results show that the respondents from different cities in Zhejiang Province have a similar preference for recyclable express packaging and green express packaging. They prefer a little more to use the latter. Respondents from Huzhou have the lowest recognition of recyclable and green express packaging among those 11 cities. The rate of their willingness for using recyclable and green express packaging is only 80.95% and 95.24%, while the awareness rate of respondents from the other 10 cities is over 85% and 96%. The respondents from Jinhua and Lishui have a higher acceptance of environment-friendly express packaging. They are more willing to use recyclable and green express packaging.

Chi-square test is conducted on the preference of consumers in different cities for recyclable and green express packages. As shown in Table 8, the significance values were 0.004 and 0.985, respectively. This shows that the urban factor has a significant impact on consumers’ choice of recyclable express packages. The reasons for this may be that consumers in different regions have different levels of acceptance of recyclable express packages. However, for the green express packages, the urban factor may have no influence on whether consumers are willing to use it.

#### 4.3.2. Cross-Cutting Relationship between Gender and Other Factors 

In terms of gender, males express more willingness to spend a higher premium of green express packaging than females. The results of chi-square test on gender and premium are shown in Table 9, *p* = 0.005 (<<0.05), that is, different genders can accept different premiums of express packaging.

The data in Table 10 show that 34.8% of females are willing to pay a premium cost that is no more than 0.5%, while the proportion of males is 26.3%, 8.52% lower than that of females. When the proportion of extra expenses increases to 0.5–1%, the number of females is 6.64% higher than that of males. If the additional cost continues to rise to more than 1%, the proportion of males ready to pay will surpass that of females. According to the statistical data, in the range of 1.01–2%, the proportion of males is 7.34% higher than that of females, and the number comes to 4.29% in the range of 2.01–5%. Up to the range of over 10%, the number of males is still higher. To sum up, males have a higher willingness to pay for green express packaging. The main reason may be that females lay more emphasis on the cost-effectiveness of online shopping.

The chi-square test results in Table 9 verified that there may be a relationship between gender and number of packages (*p* = 0.001 < 0.05). Further, we separately analyzed the monthly number of express packages received by male consumers and female consumers. As is shown in Table 11, the number of express packages received by consumers per month is most common in the range of 2 to 5. Thus, we distinguished consumers by receiving no more than five express packages monthly and receiving more than five express packages monthly. Among all respondents, 73.63% of males and 57.75% of females receive no more than five express packages per month, while 26.37% of males and 42.25% of females receive more than five express packages monthly. Therefore, it can be concluded that females receive more express packages than males do.

In addition, Table 9 shows that the chi-square test result between the frequency of online shopping and the number of express packages received is *p* << 0.05, which means that consumers with higher online shopping frequency will receive more express packages. According to the results, 0–5 times of online shopping per month applied to 70.3% of men and 55.2% of women (Table 12). Among them, people who shop online 2–5 times a month are the most, about 57.1% of male and 48.7% of female. Similarly, the consumer group who receives 2–5 express packages every month is also the largest (as shown in Table 11). About 44.8% of females indicate that they purchase online more than six times a month, compared with 29.7% of males. Regarding purchase frequency and express quantity, females buy more frequently and also receive more. Thus, compared with men, women’s awareness of environmental protection is more conducive to alleviate the environmental pressure brought by express packaging due to the more frequent purchasing.

#### 4.3.3. Summary

In this section, we mainly analyze consumers’ choice of packaging for two influencing factors (city and gender) through cross-analysis. The following results are obtained. 

(1) Respondents in different cities have similar preferences for recyclable express packaging and green express packaging, and they are more inclined to use green express packaging.

(2) The city factor only affects consumers’ choice of recyclable express packages, but does not affect whether consumers are willing to use green express packages; therefore, the city factor cannot be used to judge consumers’ preference for green express packaging. 

(3) Compared to the females, the males are more willing to pay for green express packaging, while the females maybe pay more attention to the cost-effectiveness of online shopping.

(4) Compared with the males, the females shop more frequently online and receive a correspondingly larger number of express packages. Therefore, due to their high purchasing frequency, focusing on improving women’s environmental protection awareness is more conducive to alleviating the environmental pressure caused by express packaging. 

## 5. Research Model and Testing Results

**Figure 7:**
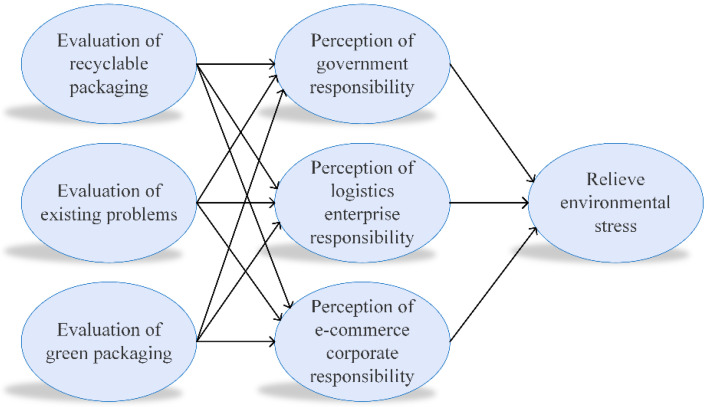
Structural equation model.

In order to promote the sustainable development of the express industry, we ought to underline the coordination between government, enterprises, and consumers [4]. Therefore, we built a sustainable development model of express packaging by the way of three-party cooperation, in which the government is the mainstay, enterprises are the main body, and consumers are the core. The model is committed to building a green supply chain system for express packaging from production to recycling, as shown in Figure 7.

In Section 4, we conduct a simple analysis of the questionnaire data. Through analysis, we intuitively understand some of the opinions and positions of consumers in the environmental pollution of express packaging. In this section, the structural equation model (SEM) is used to perform in-depth analysis of the relationship between the seven potential variables (see Figure 7).

### 5.1. Structural Equation Modeling

There are multiple relationships between the variables constructed by the questionnaire, which should be tested simultaneously. Consequently, SEM is used to perform overall verification of the model. We use SmartPLS3.0 to analyze the 561 valid data by SEM [46,47]. The model is presented in Table 13. The circles represent seven latent variables. Each latent variable derives several observed variables. There are totally 35 observable variables in the model (the item descriptions for each latent variable are shown in Supplementary Material Part S3).

### 5.2. Reliability and Validity Test

Reliability analysis refers to the reliability of the variables, see Table 13. It refers to repeatedly measuring the same target with the same method, which can be expressed by the α reliability coefficient. Cronbach’s α and composite reliability (CR) are used in our research. If the Cronbach’s α is greater than 0.7, it means the scale is considered in high reliability. Similarly, if the value of CR is greater than 0.7, it indicates that the variable is in high consistency as well [48]. The calculated results show that Cronbach’s α is greater than 0.85 and CR is greater than 0.90, which ensures the reliability of the variables. Because the two latent variables of “evaluation of recyclable express packaging” and “evaluation of green express packaging” are formative variables, the requirement of correlation between observed variables is not high. Thus, there is no combined reliability value for them. 

The validity is shown in Table 13, which indicates how well the results respond to the quality of variable measurement of the questionnaire. The greater the consistency between the measurement and examined content, the higher validity will be. Convergence validity means the similarity of the results obtained by using different measurement questions in the questionnaire to measure the same variable. If the average variance extraction (AVE) is greater than 0.5, the validity of polymerization will be higher. In these measured data, AVE is more than 0.7, which means the validity of the questionnaire is in a high level. The fact that the two variables are formed means there is no need for a high degree of correlation between variables. In other words, there is no AVE as well.

In conclusion, the results of reliability and validity test (see Table 13) show that our study is suitable for using this structural equation modeling for analysis. The analysis results of the SEM model are of great practical significance. 

### 5.3. Hypothesis Testing 

Focusing on the three aspects of government, logistics companies, and e-commerce enterprises, 12 research hypotheses in the perspective of consumers are put forward. The specific hypotheses are in Table 14. We investigate respondents’ views on some issues, such as the use of recyclable and green express packaging, the imperfect recycling mechanisms of the existing express industry, and the lack of environmental awareness of consumers. The segregation of duties among government, logistics companies, and e-commerce enterprises are also studied. Finally, from four aspects, we study whether these perceptions mentioned by respondents can help to alleviate the environmental pressure caused by express packaging. Table 14 provides the results of the hypotheses testing. 

The parameter R^2^ denotes the degree of interpretation of the external dependent latent variable on the variation of the intrinsic latent variable. In different fields, the threshold of R^2^ is not the same, which should be determined according to the research subject. Generally, the result is acceptable where the value of R^2^ is more than the critical value of 0.1. When R^2^ is around 0.19, it shows the result is in poor interpretation ability. When the value is about 0.33, it means that the model has overall rationality [49]. Based on the result shown in Table 15, the potential variables “evaluation of recyclable express packaging”, “evaluation of existing problems”, and “evaluation of green courier packaging” can explain 41.1% of “perceived to government responsibility”, 38.9% of “perceived responsibility for logistics enterprises”, and 41.6% of “e-commerce companies”, respectively. The effect of the interpretation reaches our expectations.

Meanwhile, we have tested the overall fit of our model. The overall fit for the SEM was assessed by evaluating the SRMR [50]. A value of SRMR below 0.08 indicates that a model provides a sufficient fit of the empirical data [51]. As the SRMR value for this research model was 0.059, lower than the threshold value of 0.08, it can be concluded that the model provides a good model fit.

## 6. Discussion

### 6.1. About Government 

From the results of SEM, our analysis reveals that government is supposed to take the responsibility of disposing of the pollution problems. Moreover, consumers with high environmental consciousness will contribute to the development and implementation of policies. The relevant conclusions about government are as follows.

(1) The respondents’ evaluation of recyclable express packaging has a significant positive impact on the awareness of government responsibility (*p* < 0.05). The deeper they recognize the eight characteristics of recyclable packaging, the more they understand government policies. Consumers’ recycling habits will promote construction in recycling. In other words, it has a positive impetus for the implementation of the policies.

(2) The government is one of the main forces promoting the recycling of express packaging. The respondents’ attitude towards existing problems has a positive effect on the sense of government responsibility (*p* < 0.05). Most of the respondents believe that the current problems in recycling, such as low revenue, incomplete channels, and lack of leading enterprises, make lots of consumers reluctant to take the initiative to recycle. Therefore, the relevant measures taken by the government to alleviate environmental pressures can attract the attention of most consumers and strengthen their recycling awareness.

(3) It is recommended that the government ought to issue the policies on the greening of express packaging as soon as possible. Express users’ evaluation of green express packaging has a positive impact on government responsibility (*p* < 0.05). Participants with high recognition of the eight characteristics regarding green express packaging can better understand and support the relevant policies.

### 6.2. About Logistics Companies 

There is no denying that logistics enterprises serve as a bridge between e-commerce companies and consumers, which play a pivotal role in alleviating the environmental pressure of express packaging. The following are some conclusions about logistics companies.

(1) Whether consumers are willing to use recyclable express packaging or not, logistics companies should speed up the construction of reverse logistics of packaging services. There is no significant relationship between respondents’ preference for recyclable packaging and consumers’ awareness of logistics companies’ responsibility (*p* > 0.05). In other words, consumers’ preferences for recyclable packaging do not affect the reverse logistics services of logistics enterprises. The reason for this phenomenon may be: some consumers prefer to use green express packaging, and their acceptance of recyclable express packaging is low. However, these consumers expressed the hope that logistics enterprises would speed up the construction of reverse logistics for recycling express packaging materials, so as to recover the recyclable express packaging materials in the hands of other consumers. Therefore, whether consumers can accept the recyclable express packaging or not, logistics companies should promote the construction of reverse logistics services, so as to guide consumers to use recyclable express packaging.

(2) Consumers’ cognition of existing problems can help logistics enterprises to transform and upgrade (*p* < 0.05). It is easier for logistics enterprises using environmental protection express packaging to realize sustainable development. According to the questionnaire data, consumers have a certain understanding of the existing environmental problems of express packaging, some of whom have a higher awareness of environmental protection. Logistics enterprises should seize the opportunity to change the mode from traditional express service to low-carbon logistics to meet the environmental protection needs of consumers with high environmental awareness. Moreover, there is still a large space for the majority of consumers to raise their awareness of environmental protection. Logistics enterprises using environmental protection express packaging can guide consumers with low environmental awareness to carry out low-carbon consumption and improve the latter’s environmental awareness. To sum up, logistics companies should actively recommend environmental protection express packaging, and strive to achieve their own sustainable development.

(3) The reverse service of logistics enterprises can attract consumers to use environment-friendly express packaging. The value of significance probability *p* (<0.05) indicates that consumers hope logistics companies switch to green express packaging. In fact, consumers are most concerned about the cost performance of using green express packaging. As for logistics companies, reverse service of waste express packaging can not only improve the reusability of express packaging, but also have a positive impact on environment protection. More importantly, it can cut the expenses for consumers and companies as well. 

### 6.3. About E-Commerce Companies 

In order to satisfy the business need, e-commerce companies have to use express packaging. Its industry development will continue to consume plenty of express packaging. Therefore, consumers believe that e-commerce enterprises should respond to pollution problems as soon as possible and replace the traditional packaging with environment-friendly express packaging. Based on the previous analysis, the following conclusions can be drawn. 

(1) The transforming and upgrading of express packaging can be promoted by the willingness of consumers. The respondents’ analysis of recyclable express packaging has a distinctly positive impact on e-commerce corporate responsibility awareness (*p* < 0.05). E-commerce companies can guide consumers to choose recyclable packaging by taking advantage of their preferences. Therefore, the proportion of e-commerce enterprises using recyclable packaging will vary with the acceptance of consumers. 

(2) In order to meet the business need, e-commerce companies have to use environment-friendly express packaging. Evaluation of existing problems has a significant positive influence awareness of e-commerce corporate responsibility (*p* < 0.05). The analysis shows that more than half of the respondents agree that the lack of habits and channels of recycling in daily life is one of the reasons for packaging pollution. It is not difficult to conclude that consumers’ awareness of environment protection will engage the e-commerce companies to upgrade express packaging and accelerate the elimination of traditional packaging.

(3) The environment-friendly express packaging not only meets consumers’ demand, but also enables e-commerce companies to achieve sustainable development. The index *p* (<0.05) indicates that consumers’ recognition of green packaging has a positive impact on e-commerce enterprises’ upgrading packaging. The high acceptance of green courier packaging by consumers indicates that consumers’ awareness of greening is enhanced; that is to say, consumers’ acceptance of green express packaging is not only conducive to its popularity, but also beneficial to the publicity of environmental awareness in e-commerce companies.

### 6.4. Summary 

According to the analysis of the three aspects above, the government, logistics enterprises, e-commerce companies, and consumers must cooperate to solve the problem of environmental pollution caused by express packaging.

(1) Consumers believe that the government ought to take the lead in solving the environmental pollution caused by express packaging (*p* < 0.05), while all participants should reduce environmental pollution caused by unreasonable express packaging and speed up the process of recycling and greening.

(2) Logistics companies ought to take the initiative to assume the social responsibility for environmental protection. The respondents’ awareness of logistics enterprise responsibility has a distinctly positive impact on alleviating environmental pollution pressure (*p* < 0.05). In the aspect of recycling, establishing an information system, carrying out recycling services, and providing consumers with convenience are all effective measures for logistics companies.

(3) E-commerce companies ought to shoulder the responsibility of guiding consumers to properly handle express packaging and relieve the environmental burden. The respondents’ understanding of e-commerce enterprises’ responsibility can alleviate environmental pollution pressure (*p* < 0.05). As the upstream enterprises of logistics activities, e-commerce companies should take the initiative to use environment-friendly express packaging and encourage consumers to recycle the intact packaging to reduce their damage to the environment.

## 7. Conclusions

This paper mainly demonstrates that consumers’ awareness of environmental protection is not only beneficial to reduce the pollution caused by express packaging, but also has a positive influence on improving the awareness of responsibility of government, logistics enterprises, and e-commerce companies. Although we do not study the impact of specific economic policies on environmental protection, the conclusions of the paper have reference value for the government to formulate policies in the future. The main conclusions are drawn as follows.

(1) Consumers in Zhejiang Province have a higher level of environmental awareness.

Over 80% of consumers accept the view that discarding express packaging without treatment will do damage to natural environment. About 20% of consumers express their understanding about hazards of express packaging on the environment and human beings. In Zhejiang Province, more than 95% of the consumers are willing to use recyclable and green packaging. The use of environment-friendly express packaging not only protects the environment, but also brings benefits to consumers. Considering recyclable and green express packaging will greatly reduce packaging contamination, most consumers have expressed a willingness to pay a premium to use them. There are even many people who are ready to pay an extra cost of more than 10% for the use of environment-friendly express packaging.

(2) Consumers hope the government appeals to the public to use environment-friendly express packaging.

Consumers’ awareness of environmental protection enables them to better understand the government’s policy and measures. Generally, they deem that the government should play a major role in recycling express packaging and promoting the use of green express packaging. It is suggested that the government ought to issue some tax policies, establish environmental protection funds, and encourage related research institutions to do some research to improve the environmental protection level of enterprises and reduce waste or pollution. These measures are conducive to enhancing the public awareness of environmental protection and guiding e-commerce companies and logistics enterprises to establish correct values. In addition, express packaging can be used rationally by accelerating the construction of recycling firms and forming a reasonable recycling system. The government’s advocacy of using environment-friendly express packaging is conducive for consumers to establish a correct awareness of environmental protection.

(3) Consumers hope that logistics companies and e-commerce enterprises undertake the social responsibility for environmental protection initiatively.

Consumers consider that logistics companies and e-commerce enterprises must cooperate with the government to deal with the problem of express packaging pollution as soon as possible. What is more, the waste of express packaging can be reduced by establishing recycling cooperation, using environment-friendly materials, and carrying out recycling services, etc. Actively appealing to consumers using environment-friendly express packaging is also a reflection of corporate social responsibility. Accordingly, for the sake of achieving the purpose of environmental protection, the company had better gradually use environment-friendly express packaging to replace the traditional ones. 

### 7.1. Recommendation

Based on the analysis above, we hope to establish a green supply chain system with government as the core, logistics enterprises and e-commerce companies as the two wings, and consumers as the main body. Everyone should actively respond to the pollution problem of express packaging. The following recommendations are made to the government. 

(1) Promote green express packaging in priority and give targeted promotion of recyclable express packaging.

According to the survey, consumers have a higher preference for green express packaging than recyclable express packaging. In the early stage of establishing a green market, it is better to give priority to promoting green express packaging according to the willingness of consumers. It helps to speed up the popularization of environment-friendly packaging. Further, we must conduct market research on recyclable express packaging to confirm whether consumers are willing to use it. At present, it is envisaged that some consumers will be worried about the sanitary situation of recyclable express packaging. We can recommend them to start to try from the goods that are not in direct contact with the package (such as the clothes with their own package) so that they will have some sight into recyclable express packaging in the process of using, and remove the misunderstanding of its sanitary situation. As a starting point, recyclable express packaging can be gradually promoted.

(2) Propose the tax policy for recycling and establish an environment fund promptly.

It is suggested that the government ought to introduce tax policies for express packaging to curb the abuse of non-recyclable express packaging and guide logistics enterprises, e-commerce companies, and consumers to use environment-friendly express packaging. The government should encourage logistics companies to increase a recycling mode based on the existing business modes, and establish the relevant information systems for the reverse logistics of express packaging. There is also the establishment of environmental funds to provide financial support for logistics enterprises in the early operation of the recycling system.

(3) Encourage scientific research institutions to carry out relevant research and develop environmental-friendly packaging materials. 

Herein are two suggestions for government agencies. First of all, a special fund is set up to encourage scientific research institutions to actively study new environmental protection materials and increase the application and promotion of environmental protection express packaging. Secondly, in order to achieve sustainable development of the express industry, it should be actively promoted for the cooperation between enterprises (especially logistics and e-commerce companies) and scientific research institutions in the development of new express packaging. Traditional packaging materials cannot be degraded, recycled, and reused, which will cause environmental pollution. Therefore, we hope that green raw materials can be applied in the field of express packaging as soon as possible.

(4) Strengthen the publicity of recycling packaging materials and improve the environmental protection awareness of consumers. 

It is wild cognition that disposal of express packaging will do harm to the environment or even human beings, but few people take action initiatively. The government ought to promote the concept of recycling express packaging, so that more consumers realize that express packaging should be recycled reasonably instead of being discarded directly. The governmental agencies should organize schools, communities, and village committees to carry out mass activities for the recycling express packaging. Moreover, publicizing and educating residents can be conducted in a variety of ways.

(5) The government should support leading enterprises in the recycling field to build a reverse supply chain for recycling.

Nowadays, the recycling of express packaging has not formed an industrial chain yet, which means most companies do not have recycling services for express packaging at all, and only a few enterprises or self-employed individuals are engaged in that kind of service. In other words, the scope and the influence on the recycling of express packaging are both small. Therefore, it is highly recommended that the government intervenes to promote the development of the industry through the support of resources, financials, and policies. Recycling information and resources can be integrated to expand the scale of recycling and achieve profitability.

### 7.2. Limitations and Future Prospects

In this study, the questionnaire sample of each city is representative and the proportion is consistent with the proportion of the resident population in the different cities of Zhejiang Province. However, the age distribution of the data collected in this paper is not balanced. In terms of age distribution, the number of respondents aged 21–30 is relatively large while those over 60 years old is quite small. In future studies, we will pay more attention to the age-balanced distribution of respondents. Further, more detailed and targeted questionnaires will be designed for consumers of different ages.

Secondly, our research does not investigate why consumers are worried about recyclable express packaging and why they prefer to use green express packaging. In the next study, we will combine a series of environmental policies issued by the government to analyze the consumer psychology of environmental protection express packaging more carefully, and provide reference for relevant enterprises. With the popularity of environmental protection express packaging, consumers will gradually deepen the understanding of environmental pollution caused by express packaging. Therefore, the study of consumer preference for express packaging is still one of the important research topics in the future.

## Figures and Tables

**Table 1:** Basic information of respondents.

Item		Number	Proportion
Gender	Male	252	45.5%
	Female	302	54.5%
Age	12 and below	10	1.81%
	13–20	95	17.2%
	21–30	193	34.8%
	31–40	147	26.5%
	41–50	97	17.5%
	51–60	10	1.81%
	61 and above	2	0.36%
Profession	Student	146	26.4%
	Retiree	18	3.25%
	Manufacturing practitioner	102	18.4%
	Transportation, warehousing, and postal and telecommunications practitioners	32	5.78%
	Financial industry, real estate industry, insurance industry, and commercial service industry practitioners	159	28.7%
	Others	97	17.5%
Monthly income (after tax)	Less than $547	283	51.1%
	$547–$1094	181	32.7%
	$1094–$1563	54	9.75%
	$1563–$2344	20	3.61%
	More than $2344	16	2.89%
Education	Elementary school and below	15	2.71%
	Junior high school	46	8.30%
	High school	155	28.0%
	Undergraduate	240	43.3%
	Postgraduate degree and above	98	17.7%

**Table 2:** Reliability analysis and validity analysis of Q4 and Q7.

Items	KMO	Bartlett’s χ^2^	Bartlett’s Sig.	Cronbach’s α
Q4	0.901	2165.233	0.000	0.876
Q7	0.910	2353.159	0.000	0.880

**Table 3:** The importance ranking of three factors in Question 4.

Factor	y	Ranking
Cost performance	13.8	1
Appearance	11.0	2
Environmental protection and practicality	7.71	3

**Table 4:** The importance ranking of three factors in Question 7.

Factor	y	Ranking
Cost performance	14.7	1
Appearance	11.8	2
Environmental protection and practicality	7.90	3

**Table 5:** Respondents’ awareness of government responsibility.

Questions/Options	Totally Disagree	A Little Disagree	Uncertain	Somewhat Agree	Totally Agree
Introduced a tax policy	2.26%	3.99%	18.9%	52.3%	22.6%
Set up environmental fund	1.56%	4.51%	19.1%	52.2%	22.6%
Develop new materials	1.56%	3.99%	19.1%	50.7%	24.7%
Enhance publicity on recycling	2.26%	4.17%	20.0%	49.0%	24.7%

**Table 6:** Respondents’ awareness of logistics corporate responsibility.

Questions/Options	Totally Disagree	A Little Disagree	Uncertain	Somewhat Agree	Totally Agree
Reduce the use of plastic tape	1.56%	4.17%	17.4%	51.2%	25.7%
Open the door-to-door recycling services	1.91%	3.82%	17.7%	50.0%	26.6%
Guide customers to use recyclable or green packaging	2.26%	3.30%	20.8%	50.4%	23.3%
Establish information systems for recycling	1.91%	2.95%	18.1%	52.4%	24.7%

**Table 7:** Respondents’ awareness of e-commerce corporate responsibility.

Questions/Options	Totally Disagree	A Little Disagree	Uncertain	Somewhat Agree	Totally Agree
Promise to use recyclable or green packaging	1.74%	4.34%	17.9%	53.5%	22.6%
Establish recycling cooperation with logistics companies	1.56%	3.65%	17.5%	53.3%	24.0%
Reward consumers for recycling	1.74%	2.78%	15.6%	54.7%	25.2%

**Table 8:** Table of chi-square test results on urban.

	Value	Exact Sig. (2 Sided)
Fisher’s exact test for city’s recyclable express packaging	22.9	0.004
Fisher’s exact test for city’s green express packages	3.24	0.985

**Table 9:** Table of chi-square test results on gender and other factors.

	Value	Sig.
Continuity correction between gender and premium	17.0	0.005
Pearson chi-square between gender and number of packages	19.5	0.001
Pearson chi-square between the frequency of online shopping and the number of express packages	635.2	0.000

**Table 10:** Regarding the premium of green express packaging, the relationship between gender and acceptance.

Item	<0.5%	0.5–1%	1.01–2%	2.01–5%	5.01–10%	>10%
Male	26.3%	32.7%	22.2%	12.0%	5.64%	1.13%
Female	34.8%	39.4%	14.8%	7.74%	2.26%	0.97%

**Table 11:** Relationship between gender and number of express packages in a month.

Quantity/Month	0–1	2–5	6–10	11–15	16 or More
Male	13.9%	59.8%	18.1%	4.51%	3.76%
Female	8.39%	49.4%	28.7%	8.39%	5.16%

**Table 12:** Relationship between gender and frequency of shopping online.

Item	0–1	2–5	6–10	11–15	16 or More
Male	13.2%	57.1%	15.8%	8.65%	5.26%
Female	6.45%	48.7%	30.0%	8.39%	6.45%

**Table 13:** Results of reliability and validity tests.

Variables	Cronbach’s α	rho_A	CR	AVE
Evaluation of recyclable packaging	0.884	1.000		
Evaluation of existing problems	0.859	0.877	0.905	0.706
Evaluation of green packaging	0.894	1.000		
Awareness of government responsibility	0.885	0.887	0.921	0.743
Awareness of logistics enterprise responsibility	0.892	0.893	0.925	0.756
Awareness of e-commerce corporate responsibility	0.859	0.860	0.914	0.780
Relieve environmental pressure	0.910	0.911	0.937	0.789

**Table 14:** Result of hypotheses testing.

Assumed Content	Hypothetical Result	*p* Value	Result
Evaluation of recyclable express packaging → Awareness of government responsibility (+)	Positive effect	7.99 × 10^−3^	Support
Evaluation of existing problems → Awareness of government responsibility (+)	Positive effect	4.06 × 10^−11^	Support
Evaluation of green courier packaging → Awareness of government responsibility (+)	Positive effect	5.28 × 10^−4^	Support
Evaluation of recyclable express packaging → Awareness of logistics enterprise responsibility (+)	Not Significant	1.16 × 10^−1^	Nonsupport
Evaluation of existing problems → Awareness of logistics enterprise responsibility (+)	Positive effect	6.65 × 10^−12^	Support
Evaluation of green courier packaging → Awareness of logistics enterprise responsibility (+)	Positive effect	1.68 × 10^−3^	Support
Evaluation of recyclable express packaging → Awareness of e-commerce corporate responsibility (+)	Positive effect	4.71 × 10^−3^	Support
Evaluation of existing problems → Awareness of e-commerce corporate responsibility (+)	Positive effect	5.68 × 10^−14^	Support
Evaluation of green courier packaging → Awareness of e-commerce corporate responsibility (+)	Positive effect	4.99 × 10^−3^	Support
Awareness of government responsibility → Relieve environmental pressure (+)	Positive effect	1.85 × 10^−6^	Support
Awareness of logistics enterprise responsibility → Relieve environmental pressure (+)	Positive effect	3.64 × 10^−2^	Support
Awareness of e-commerce corporate responsibility → Relieve environmental pressure (+)	Positive effect	2.18 × 10^−2^	Support

‘+’ indicates positive correlation.

**Table 15:** R^2^ test of four latent variables.

Item	R^2^	Adjusted R^2^
Awareness of government responsibility	0.411	0.407
Awareness of logistics enterprise responsibility	0.389	0.385
Awareness of e-commerce corporate responsibility	0.416	0.413
Relieve environmental pressure	0.389	0.385

## Data Availability

All data generated or analyzed during this study are available from the corresponding author upon reasonable request.

## Supplementary Materials

The following supporting information can be downloaded at: https://www.mdpi.com/article/10.3390/ijerph19084895/s1.
