# Supplementary material for: Consumer Cognition and Management Perspective on Express Packaging Pollution
Source: Int J Environ Res Public Health. 2022 Apr 18;19(8):4895. doi: 10.3390/ijerph19084895 (PMC9029519; doi:10.3390/ijerph19084895)
Supplement: Supplementary file 1 [file ijerph-19-04895-s001.zip › ijerph-1616429-supplementary.pdf]

Supplementary Materials

Consumer Cognition and Management Perspective on Express Packaging Pollution

Sisi Wu [1], Xuan Gong [1], Yunfei Wang [1,*] and Jian Cao [1,2]

1 School of Management, Zhejiang University of Technology, Hangzhou 310023, China; sswu_zjut@163.com (S. W.); gongxuan_up@163.com (X. G.); yfwang_zjut@163.com (Y. W.);jcao@zjut.edu.cn (J. C.)

2 Center for Global & Regional Environmental Research, the University of Iowa, Iowa City 52242, United States

** Correspondence: yfwang_zjut@163.com*

Part S1

- ***Questionnaire design***

Likert five-level scale is used to evaluate the questions in our questionnaire. A pre-survey which includes 57 participants is used to avoid the ambiguity and increase the validity of the questionnaire. The initial questionnaire is revised and improved based on the opinions and suggestions provided by the participants. While the formal questionnaire covering all age groups in Zhejiang Province was released on April 19, 2019.

In order to facilitate the analysis, we divided the 20 questions into two parts, namely the basic information part and other parts. The former includes seven questions, corresponding to questions 14 to 20 of the questionnaire. While the latter is the main content of the questionnaire, corresponding to questions 1 to 13.

Part S2

Part S2.1

- ***Factor analysis of recyclable express packaging***

As is shown in **Error! Reference source not found.**, three factors are extracted from Question 4, which explain the "75.6% of the total variance". In general, it is acceptable when the total variance is greater than 70% in social science surveys. So we extract three factors totally.

Table S1. Total variance explained of Q4.

Component	Initial Eigenvalue			Extraction Sums of Squared Loadings			Rotation Sums of Squared Loadings		
	Total	% of Variance	Cumulative %	Total	% of Variance	Cumulative %	Total	% of Variance	Cumulative %
1	4.522	56.524	56.524	4.522	56.524	56.524	2.739	34.241	34.241
2	0.880	10.995	67.519	0.880	10.995	67.519	1.971	24.643	58.884
3	0.643	8.040	75.559	0.643	8.040	75.559	1.334	16.675	75.559
4	0.526	6.576	82.135						
5	0.438	5.472	87.608						
6	0.390	4.874	92.482						
7	0.340	4.248	96.729						
8	0.262	3.271	100.000						

Extraction Method: Principal Component Analysis.

The eight elements involved in Question 4 (appearance, price, reusability, firmness, convenience

of use, environmental pollution, convenience of recycling and return on recycling) are summarized as three factors of 'environmental protection and practicality', 'cost performance' and 'appearance'. As we can see from Table S1, Factor 1 (environmental protection and practicality) corresponds to elements 4.5 to 4.8. Factor 2 (cost performance) corresponds to elements 4.1, 4.3 and 4.4. Factor 3 (appearance) corresponds to element 4.2.

Table S2. Rotated structure matrix[a] of Q4.

	Component		
	1	2	3
4.2 Appearance	0.131	0.227	0.889
4.1 Price	0.156	0.754	0.383
4.3 Reusability	0.428	0.695	0.251
4.4 Firmness	0.474	0.754	0.001
4.5 Convenience of use	0.785	0.304	0.112
4.6 Degree of environmental pollution	0.790	0.296	0.047
4.7 Convenience of recycling	0.791	0.309	0.235
4.8 Return on recycling	0.652	0.153	0.513

Extraction Method: Principal Component Analysis.
Rotation Method：Varimax with Kaiser Normalization.
a Rotation converged in 6 iterations.

For the sake of studying the consumers' attention to these three factors, principal component analysis is used to analyze question 4, calculate the extracted three factors by score of principal component, and take the calculated results as the basis of the importance ranking of corresponding factors. The weight of each formula is the ratio of the eigenvalue of each element to the arithmetic square root of the eigenvalue of its principal component. The principal component model of these three factors is as follows:

$$y_1=0.062x_1+0.073x_2+0.201x_3+0.223x_4+0.369x_5+0.372x_6+0.372x_7+0.307x_8$$

$$y_2=0.242x_1+0.804x_2+0.741x_3+0.804x_4+0.324x_5+0.316x_6+0.329x_7+0.163x_8$$

$$y_3=1.109x_1+0.478x_2+0.313x_3+0.001x_4+0.140x_5+0.059x_6+0.293x_7+0.640x_8$$

The data from561 questionnaires were put into the model for calculation. The value of y in Table S3 is the quantitative value of the importance of these three factors to consumers, which is obtained by averaging the corresponding values of all questionnaire data.

Table S3. The importance ranking of Q4.

Factor	y	Ranking

Cost performance	13.8	1
Appearance	11.0	2
Environmental protection and practicality	7.71	3

Part S2.2

- ***Factor analysis of green express packaging***

Analysis of question 7 further, as shown in the Table S3, three factors can be extracted to explain the content of over 70% (76.9%) of the total variance. So we extract all three factors as well.

Table S4. Total variance explained of Q7.

Component	Initial Eigenvalue			Extraction Sums of Squared Loadings			Rotation Sums of Squared Loadings		
	Total	% of Variance	Cumulative %	Total	% of Variance	Cumulative %	Total	% of Variance	Cumulative %
1	4.716	58.950	58.950	4.716	58.950	58.950	2.861	35.757	35.757
2	0.853	10.657	69.607	0.853	10.657	69.607	2.014	25.174	60.931
3	0.584	7.295	76.902	0.584	7.295	76.902	1.278	15.971	76.902
4	0.467	5.831	82.733						
5	0.439	5.489	88.222						
6	0.365	4.568	92.790						
7	0.303	3.784	96.574						
8	0.274	3.426	100.000						

Extraction Method: Principal Component Analysis.

The eight elements involved in Question 7 are the same as those in question 4, which can also be summarized into three factors, as shown in Table S5 and Table S6. Factor 1 corresponds to elements 7.5 to 7.8, called environmental protection and practicality. Factor 2 corresponds to elements 7.1, 7.3 and 7.4, called cost performance. Factor 3 corresponds to element 7.2, called appearance.

Table S5. Rotated structure matrix[a] of Q7.

	Component		
	1	2	3
7.2 Appearance	0.136	0.251	0.916
7.1 Price	0.205	0.851	0.244

7.3 Reusability	0.484	0.574	0.395
7.4 Firmness	0.513	0.662	0.164
7.5 Convenience of use	0.705	0.441	0.130
7.6 Degree of environmental pollution	0.724	0.428	-0.027
7.7 Convenience of recycling	0.824	0.268	0.129
7.8 Return on recycling	0.777	0.096	0.402

Extraction Method: Principal Component Analysis.

Rotation Method：Varimax with Kaiser Normalization.

a Rotation converged in 6 iterations.

Similarly, after the same principal component analysis as question 4, the importance ranking of the three factors of Q7 can be obtained. The principal component model is shown below:

$$y_1=0.063x_1 + 0.094x_2 + 0.223x_3 + 0.236x_4 + 0.325x_5 + 0.333x_6 + 0.379x_7 + 0.358x_8$$

$$y_2=0.272x_1 + 0.921x_2 + 0.621x_3 + 0.717x_4 + 0.477x_5 + 0.463x_6 + 0.290x_7 + 0.104x_8$$

$$y_3=1.199x_1 + 0.319x_2 + 0.517x_3 + 0.215x_4 + 0.170x_5 - 0.035x_6 + 0.169x_7 + 0.526x_8$$

The value of y in Table 6 is the quantitative value of the importance of these three factors to consumers, which is obtained by averaging the corresponding values of all questionnaire data.

Table S6. The importance ranking of Q7.

Factor	y	Ranking
Cost performance	14.7	1
Appearance	11.8	2
Environmental protection and practicality	7.90	3

Part S3

The structural equation model used in this paper is shown in Fig. S1. Eight observation variables are used to test consumers' evaluation of recyclable express packaging and green express packaging. These eight observation variables correspond to the eight questions in the questionnaire, namely, price, appearance, reusability, firmness, convention of using, environmental pollution, convention of recycling and return on recycling. Consumers' cognition of existing problems is tested from four aspects, corresponding to question 5 in the questionnaire. The observation variables of consumers' perception of the responsibility of the government, logistics enterprises and e-commerce enterprises correspond to the 11th question of the questionnaire. Finally, we take the four questions of question 9 as observation variables to study whether it can alleviate the environmental pressure caused by express packaging.

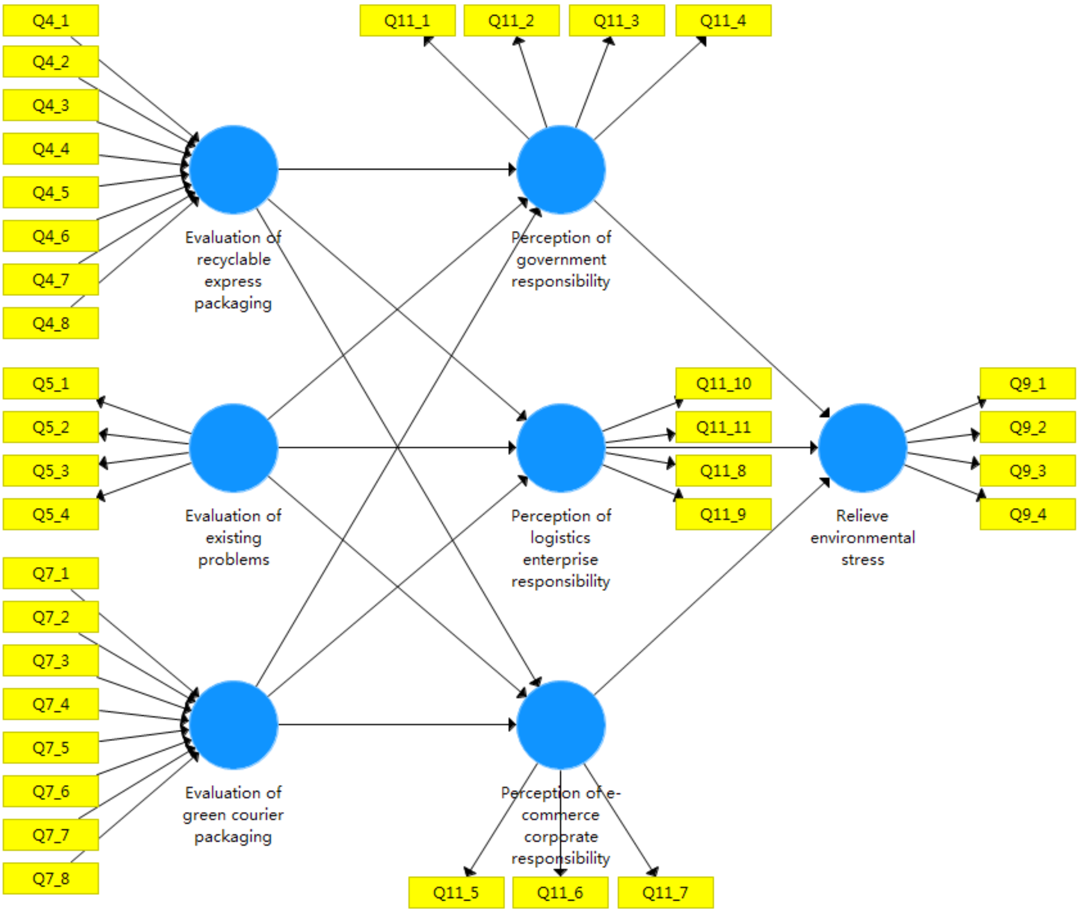

Figure. S1 SEM details diagram.

Appendix A

Questionnaire on the environmental pollution of express packaging

Hello, dear madam / sir! I am a graduate student from the School of Management of Zhejiang University of Technology. I am doing a research on environmental pollution caused by express packaging. Can you please fill out the following questionnaire? Your identity and answers are absolutely confidential and will not be used for any commercial purposes. There is no right or wrong question option, please fill in according to your actual situation. Thank you for your support and help!

1. Have you ever used the express delivery service before?

□Yes **□No**

2. Do you know the environmental pollution caused by express packaging?

	Completely	Basically	Know	Basically	Completely

	don’t know	don’t know	a little	know	Know
2.1 In the process of producing inferior plastic bags, tapes and cushions, heavy metals and inorganic gases that cause respiratory and blood diseases are emitted					
2.2 Inferior packaging materials are not degradable or difficult to degrade, and contaminated land and groundwater resources after landfill					
2.3 Inferior packaging materials are not degradable or difficult to degrade, contaminated land and groundwater resources after landfill					

3. Would you like to use **recyclable express packaging**? (Recyclable express packaging refers to plastic express boxes, shared express boxes, plastic cushions, etc. that can be recycled.)

□ Yes, I would like to. □ No, I would not like to.

4. Do you care about the following characteristics of **recyclable express packaging**? (Recyclable express packaging refers to plastic express boxes, shared express boxes, plastic cushions, etc. that can be recycled.)

	Don't care at all	Don't care	Not sure	Care about	Care about extremely
4.1 Price					
4.2 Appearance					
4.3 Reusability					
4.4 Firmness					
4.5Convenience of Using (For example: easy to remove)					
4.6 Environmental Pollution					
4.7Convenience of Recycling					
4.8 Return on Recycling (For example: recovering the return of money)					

5. What is your recognition of the following phenomena?

	Completely disagree	Tend to disagree	Not sure	Tend to agree	Completely agree
5.1 The low level of consumer willingness in recycling because					

of the low return in recycling<br>5.2 The environmental awareness of consumers need to be improved<br>5.3 Lacking flexibility and diversity in recycling channel<br>5.4 Lacking leading companies in recycling					

6. Would you like to use **green courier packaging**? (Green express packaging refers to degradable carton, degradable tape, etc.)

□Yes, I would like to. □No, I would not like to.

7. Do you care about the following features of **green express packaging**? (Green express packaging refers to degradable carton, degradable tape, etc.)

	Don't care at all	Don't care	Not sure	Care about	Care about extremely
7.1 Price					
7.2 Appearance					
7.3 Reusability					
7.4 Firmness					
7.5 Convenience of Using（For example: easy to remove）					
7.6 Environmental Pollution					
7.7 Convenience of Recycling					
7.8 Return on Recycling (For example: recovering the return of money)					

8. The psychological **maximum price** you are willing to pay for green packaging is the ____ of the commodity price?

□<0.5% □0.5%-1% □1.01%-2% □2.01%-5% □5.01%-10% □>10%

9. If recycling mechanism in china is increasingly perfect, and green express packaging are widely used, do you agree with the following behaviors as ____?

	Completely disagree	Tend to disagree	Not sure	Tend to agree	Completely agree
9.1 Willing to accept a simple package of express delivery					
9.2 Willing to use recyclable or green express delivery packaging initiatively					
9.3 Willing to participate in the					

recycling activities of express packaging initiatively					
9.4 Willing to recycle as much express packaging as possible					

10. Who should take the responsibility for managing or reducing the environmental pollution caused by express packaging?

□Government □E-commerce company □Logistics company □Consumer

11. Do you think the following measures can alleviate the environmental pollution caused by express packaging?

	Completely disagree	Tend to disagree	Not sure	Tend to agree	Completely agree
11.1 Introduce the tax policy by government promptly to reduce the pollution of express packaging					
11.2 The government set up an environmental fund to help companies establish a scientific recycling system					
11.3 The government guides the establishment of scientific research institutions to promote research on environment-friendly materials					
11.4 The government strengthens the promotion of the recycling of express packaging					
11.5 Leading e-commerce companies promise to use recyclable or green express packaging					
11.6 E-commerce companies and logistics companies establish recycling cooperation					
11.7 E-commerce companies reward consumers for recycling					
11.8 Logistics companies try to use non-adhesive cartons or paper tape to reduce the use of plastic tape					
11.9 Logistics companies open door-to-door recycling packaging services					
11.10 Logistics companies guide customers to use recyclable or green					

courier packaging
11.11 Logistics companies establish information systems for recycling express packaging

12. Your online shopping frequency (an average of one month) is probably ____?
□0-1 times per month □2-5 times per month □6-10 times per month
□10-15 times per month □16 times or more per month

13. How many express packaging do you receive per month?
□0-1 □2-5 □6-10 □11-15 □16 or more

14. [**Multiple choices**] Which three types of packaging do you think are a large amount of waste in daily life?
□Waybill □Envelope □Carton □Plastic bag □Woven bag □Plastic tape □Cushion

15. What is your gender?
□Male □Female

16. How old are you?
□12 and under □Between 13 and 20 □Between 21 and 30
□Between 31 and 40 □Between 41 and 50 □Between 51 and 60
□61 and above

17. What is your occupation?
□Student □Retiree □Manufacturing practitioner
□Transportation, warehousing and postal and telecommunications practitioners
□Financial industry, real estate industry, insurance industry and commercial service industry practitioners
□others____________________

18. How much is your gross salary?
□Less than USD 547 □Between USD 547 and 1,094
□Between USD 1,094 and 1,563 □Between USD 1,563 and 2,344
□More than USD 2,344

19. What is your highest education?
□Elementary school and below □Junior high school □High school
□Undergraduate □Postgraduate degree and above

20. Where is your current location?
□Hangzhou □Ningbo □Huzhou □Wenzhou □Jiaxing □Jinhua
□Lishui □Quzhou □Shaoxing □Taizhou □Zhoushan
□Others____________________

The above is the entire content of this questionnaire, thank you for your cooperation!

Filename: ijerph-1616429-supplementary-1.docx
Directory: E:\4.18\ijerph-1625593
Template: C:\Users\MDPI\AppData\Roaming\Microsoft\Templates\Normal.dotm
Title:
Subject:
Author: lhy
Keywords:
Comments:
Creation Date: 4/14/2022 4:46:00 PM
Change Number: 3
Last Saved On: 4/18/2022 2:20:00 PM
Last Saved By: MDPI
Total Editing Time: 0 Minutes
Last Printed On: 4/18/2022 2:20:00 PM
As of Last Complete Printing
Number of Pages: 11
Number of Words: 2,307 (approx.)
Number of Characters: 12,833 (approx.)
